# A Case of Crohn’s Disease with Cardiac Tamponade Caused by Tuberculous Pericarditis: Assessment of a Rare Phenomenon

**DOI:** 10.3390/healthcare9060695

**Published:** 2021-06-09

**Authors:** Keiichi Tominaga, Takanao Tanaka, Mimari Kanazawa, Shoko Watanabe, Rena Nemoto, Keiichiro Abe, Akira Kanamori, Akira Yamamiya, Kenichi Goda, Yoshitomo Kushima, Kazuyuki Chibana, Taito Masawa, Tomohiro Fukuda, Toshifumi Hibi, Atsushi Irisawa

**Affiliations:** 1 Department of Gastroenterology, Dokkyo Medical University, Tochigi 321-0293, Japan; tana1986@dokkyomed.ac.jp (T.T.); mimari77@dokkyomed.ac.jp (M.K.); shoko-t@dokkyomed.ac.jp (S.W.); rena-n@dokkyomed.ac.jp (R.N.); abe9841@dokkyomed.ac.jp (K.A.); k-akira@dokkyomed.ac.jp (A.K.); akira-y@dokkyomed.ac.jp (A.Y.); goda@dokkyomed.ac.jp (K.G.); irisawa@dokkyomed.ac.jp (A.I.); 2 Department of Pulmonary Medicine and Clinical Immunology, Dokkyo Medical University, Tochigi 321-0293, Japan; kushima@dokkyomed.ac.jp (Y.K.); kchibana@dokkyomed.ac.jp (K.C.); 3 Department of Cardiovascular Medicine, Dokkyo Medical University, Tochigi 321-0293, Japan; taito-ea@dokkyomed.ac.jp; 4 Center for Advanced IBD Research and Treatment, Kitasato University Kitasato Institute Hospital, Tokyo 108-8642, Japan; t.fukuda92@gmail.com (T.F.); thibi@insti.kitasato-u.ac.jp (T.H.); 5 Department of Gastroenterology and Hepatology, Kitasato University Kitasato Institute Hospital, Tokyo 108-8642, Japan

**Keywords:** Crohn’s disease, cardiac tamponade, tuberculous pericarditis, ustekinumab, *Mycobacterium tuberculosis*

## Abstract

A 28-year-old woman was hospitalized for cardiac tamponade caused by tuberculous pericarditis. She was taking ustekinumab (UST) for Crohn’s disease. UST is not considered to significantly increase the risk of developing serious infections, including tuberculosis. However, there is still a risk of *Mycobacterium tuberculosis* reactivation. Therefore, for patients on concurrent UST and antituberculosis medication, a close collaboration among specialists in infectious diseases, cardiology, and gastroenterology is necessary.

## 1. Introduction

Despite the reduced numbers of tuberculosis patients in Japan, a World Health Organization (WHO) definition of the “pre-elimination” for tuberculosis (less than 10% per 100,000 population) has not been achieved [1]. Patients with inflammatory bowel disease (IBD) who are treated with immunosuppressive therapies such as corticosteroids, antitumor necrosis factor-α (TNF-α) antibodies, and Janus kinase inhibitors need to be mindful of possibly developing intrapulmonary tuberculosis or extrapulmonary tuberculosis, such as tuberculous pericarditis. We present a patient with Crohn’s disease (CD) on ustekinumab (UST) who developed cardiac tamponade caused by tuberculous pericarditis. Latent tuberculosis reactivation is extremely rare, and there have been no reported cases with cardiac symptoms that were taking UST.

## 2. Case Report

**Figure 1:**
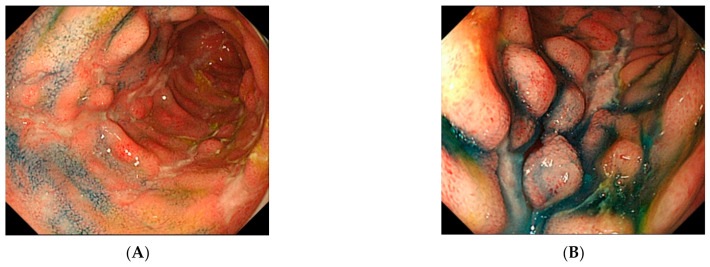
Enteroscopic findings with indigo carmine dye-sprayed images. Longitudinal ulcer (**A**) and cobblestone appearance (**B**).

A 28-year-old woman had persistent diarrhea since September 201X. Eleven months later, she was aware of a hemorrhoidal fistula and visited our hospital. After balloon-assisted enteroscopy, she was diagnosed with stricturing ileocolonic CD, Montreal classification A2L3B2p (Figure 1). At the time of diagnosis, the endoscopic inflammation was severe, and the Crohn’s Disease Activity Index was 256. Although chest and abdominal computed tomography (CT) showed no evidence of active tuberculosis, the interferon-γ (IFN-γ) release assay and tuberculin reaction were positive. The patient did not have the epidemiological risk factors for tuberculosis, such as contact with infected patients or trips to risk areas. However, considering the risk of tuberculosis, prednisolone (PSL) 40 mg/day was given to induce remission, together with isoniazid (INH) 300 mg/day. The patient’s condition improved temporarily but relapsed during PSL tapering, and adalimumab (ADA) was given. We considered and rejected administering mesalamine, as the ADA showed efficacy. However, the patient gradually developed a loss of response to ADA and was switched to infliximab (IFX) and azathioprine (AZA).

The patient maintained their remission with IFX and AZA. However, nine months after IFX, AZA, and INH coadministration, her fever persisted, and a computed tomography (CT) showed lymph node swelling in the mediastinum and hilar regions. The polymerase chain reaction (PCR) test of endobronchial ultrasound-guided trans-bronchial needle aspiration was positive for *Mycobacterium tuberculosis*, and the patient was diagnosed with extrapulmonary tuberculosis and tuberculous lymphadenitis. Consequently, IFX was discontinued, and the patient was treated with INH 300 mg/day, rifampicin (RIF) 450 mg/day, ethambutol hydrochloride (EB) 750 mg/day, and pyrazinamide (PYR) 1.5 g/day. The tuberculosis was considered resolved based on symptom improvement and CT findings, but the abdominal pain, diarrhea, aphthous stomatitis, and peri-anal pain worsened. A relapse of Crohn’s disease was suspected, and IFX was restarted with combined ISO and RIF. However, she immediately had an IFX-induced infusion reaction (nausea, vomiting, and rash) and was again switched to ADA. ISO and RIF were continued for one year. ADA was continued, with a gradual loss of response until it eventually became ineffective. ADA was then changed to UST.

**Figure 2:**
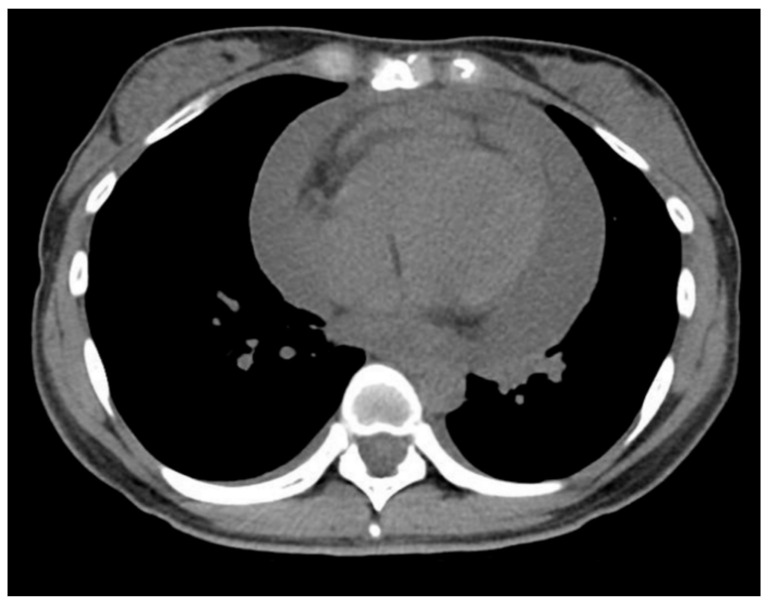
A computed tomography showing pericardial effusion.

Thirty-one months after UST, the patient had a re-exacerbation of their abdominal symptoms, and a balloon-assisted enteroscopy was performed. Enteroscopy showed severe inflammation and stenosis at the terminal ileum. Thirty-four months after UST, ileocecal resection was performed due to the difficulty of endoscopic treatment such as balloon dilatation. After surgery, remission was maintained by UST. However, four months after surgery and 38 months after the switch from ADA to UST, the patient relapsed with malaise, fatigue, watery diarrhea (five times/day), and fever. On admission, she was alert, temperature was 37.6 °C, blood pressure was 120/72 mmHg, and pulse rate was 122 beats per minute. In addition, the patient had a paradoxical pulse and jugular venous distension. The laboratory data showed the following: white blood cells 6500/μL, hemoglobin 10.9 g/dL, platelets 30.7 × 10^4^/μL, albumin 3.6 g/dL, C-reactive protein 7.74 mg/dL, erythrocyte sedimentation rate 57 mm/h, brain natriuretic peptide 67.3 pg/mL, and D-dimer 4.0 μg/mL (Table 1). Electrocardiography showed no ST segment change. Transthoracic echocardiography showed pericardial effusion, right atrial collapse, and no thrombus or shunt in the left atrium or left ventricle. Chest CT also showed pericardial effusion, and the patient was therefore diagnosed with pericardial tamponade (Figure 2). The patient immediately underwent emergency pericardiocentesis drainage. About 500mL of slightly bloody pericardial effusion was drained. Table 2 shows the pericardial effusion examination results. The pericardial effusion was found to be exudative, negative for the antimicrobial culture, smear, and TB-PCR, with high adenosine deaminase (118 U/L). Tuberculous pericarditis was diagnosed. The patient was treated with four antituberculosis drugs concurrently (ISO 300 mg/day, RIF 450 mg/day, EB 750 mg/day, and streptomycin (SM) 1000 mg (intramuscular injection three times a week)), while UST and AZA were discontinued. The patient’s symptoms improved, and she was discharged on the 15th hospital day without pericardial effusion re-accumulation. The patient’s CD tended to relapse when UST and AZA were discontinued. Therefore, UST was resumed, together with the four antituberculosis drugs.

## 3. Discussion

In general, macrophages phagocytose and digest bacteria during an infection. However, *Mycobacterium tuberculosis* is an intracellular parasite and survives in macrophages even after phagocytosis [2]. Macrophage activation, such as by TNF-α and IFN-γ, usually induces a bactericidal action in infected cells. In addition, macrophages stimulated by *Mycobacterium tuberculosis* infection produce interleukin (IL)-12, which induces Th0-to-Th1 differentiation. Th1 cells, in turn, produce IFN-γ, which activates macrophages to destroy bacteria [3,4,5]. Theoretically, IL-12/23, p40, and TNF-α inhibitors could increase the risk of *Mycobacterium tuberculosis* reactivation. Although AZA is an immunomodulatory drug, a study of the IBD cases before IFX suggests a very low risk of AZA-associated tuberculosis [6].

The etiologies of acute pericarditis, other than idiopathic pericarditis, are uremia, infection, post-myocardial infarction, rheumatoid, and malignancy, whereas tuberculous pericarditis is rare. In acute pericarditis with cardiac tamponade, Jain et al. reported that 60% of cases were tuberculous [7], and Permanyer-Miralda et al. reported 7% [8]. While these differences may be attributed to differing regional prevalences, they clearly indicate that tuberculosis can cause acute pericarditis.

The main symptoms of tuberculous pericarditis are dyspnea, fever, cough, chest pain, and general malaise [9]. The present case showed some of these symptoms. Tuberculous pericarditis is treated with antituberculosis drugs. However, when there is a massive epicardial effusion, especially with cardiac tamponade present, pericardiocentesis is required. In some cases, surgical procedures such as epicardial window placement are required [10,11].

UST is a humanized monoclonal anti-IL-12/23 antibody. UST targets p40, a common subunit of IL-12 and IL-23, and is effective for treating chronic inflammatory diseases such as psoriasis, CD, and ulcerative colitis. IL-12 promotes Th1 differentiation, whereas IL-23 promotes Thl7 differentiation. Consequently, UST suppresses both Th1 and Th17 differentiation [12]. The effectiveness of UST in induction and maintenance therapy for CD was validated in a large clinical trial (UNITI study) [13]. In this case, UST was selected as the third biological agent, because the patient also had a strong infusion reaction or resistance to two anti-TNF-α antibodies. For UST-associated tuberculosis, a case of suspected active primary tuberculosis was previously reported in a patient with CD 10 months after the patient’s last subcutaneous injection [14]. Only one patient who received UST during the PEARL large clinical trial for psoriasis in Korea and Taiwan had a latent tuberculosis reaction [15]. Furthermore, an analysis of the Korean national database reported that UST does not increase the risk of tuberculosis [16], and extensive clinical data suggests that UST does not increase the risk of developing serious infectious diseases, including tuberculosis.

In the present case, a 9-month regimen for tuberculosis was administered, consisting of four drugs (INH, RIF, EB, and SM) for two months, followed by two drugs (INH and RIF) for seven months. This 9-month regimen is recommended for patients with tuberculosis taking anti-TNF-α agents. This regimen takes into account the treatment response rate and avoids the risk of resistant strains.

In the present patient, tuberculosis did not flare up despite continued treatment with biological agents. This may have resulted from continual TNF-α production due to persistent inflammation, which countered the reactivation effect of the biological agents on *Mycobacterium tuberculosis*. We hypothesize that surgical resection of the inflammatory bowel decreased TNF-α, and consequently, UST administration resulted in *Mycobacterium tuberculosis* reactivation and tuberculous pericarditis. However, we do not have any supporting data, and this hypothesis is completely speculative.

The present patient is currently taking antituberculosis medication while continuing UST. It is important for specialists in infectious diseases, cardiology, and gastroenterology to collaborate closely to determine the treatment after antituberculosis medication is discontinued. The risk of tuberculosis recurrence must be continually assessed so that the treatment can be optimized while preventing a postoperative recurrence. Moreover, in general, when treating IBD patients with latent tuberculosis, great care must be taken to avoid tuberculosis reactivation, regardless of the use of anti-TNF-α antibodies.

## Figures and Tables

**Table 1:** Laboratory data on admission.

AST	16 U/L	WBC	6500/μL
ALT	14 U/L	RBC	3.75 × 1012/L
LDH	197 U/L	Hb	10.9 g/dL
UN	5.0 mg/dL	Plt	30.7 × 104/μL
Cre	0.46 mg/dL		
Na	141 mmol/L	ESR (1h)	57 mm/h
K	3.4 mmol/L		
Cl	103 mmol/L	D-dimer	4.0 μg/mL
TP	5.2 g/dL		
Alb	3.6 g/dl	BNP	67.3 pg/mL
CRP	7.74 mg/dL		

Abbreviations: AST: Aspartate aminotransferase; ALT: Alanine aminotransferase; LDH: Lactate dehydrogenase; UN: Urea nitrogen; Cre: Creatinine; Na: Sodium; K: Potassium; Cl: Chloride; TP: Total protein; Alb: Albumin; CRP: C-reactive protein; WBC: White blood cell; RBC: Red blood cell; Hb: Hemoglobin; Plt: Platelet; ESR: Erythrocyte sedimentation rate; BNP: Brain natriuretic peptide.

**Table 2:** Laboratory data at the onset of cardiac tamponade.

LDH	920 U/L	WBC	4600/μL	Adenosine deaminase	118 U/L
TP	5.2 mg/dL	NEUTRO	20.1%	Hyaluronic acid	74900 ng/mL
Alb	2.9 mg/dl	EOSINO	0.3%	CEA	0.5 ng/mL
Na	142 mmol/L	BASO	1.5%	TPA	6277 U/L
K	2.9 mmol/L	MoC	8.1%		
Cl	107 mmol/L	LYMPHO	70.0%	PCR—Tb	negative
AMY	40 U/L	RBC	0.55 × 10^12^/L	PCR—M. avium	negative
Glu	60 mg/dL	Hb	1.5 g/dL	PCR—M. intracellulare	negative
		Plt	1.2 × 10^4^/μL		

Abbreviations: LDH: Lactate dehydrogenase; TP: Total protein; Alb: Albumin; Na: Sodium; K: Potassium; Cl: Chloride; AMY: Amylase; Glu: Glucose; WBC: White blood cell; NEUTRO: Neutrophil; EOSINO: Eosinophil; BASO: Basophil; MoC: Monocyte; LYMPHO: Lymphocyte; RBC: Red blood cell; Hb: Hemoglobin; Plt: Platelet; CEA: Carcinoembryonic antigen; TPA: Tissue polypeptide antigen; PCR: Polymerase chain reaction; Tb: Tuberculosis; M. avium: *Mycobacterium avium*; M. intracellulare: *Mycobacterium intracellulare*.

## Data Availability

Not applicable.
